# Vulnerability Assessment and Application of Bacterial Technology on Urban Rivers for Pollution Eradication

**DOI:** 10.1155/2015/241769

**Published:** 2015-10-01

**Authors:** Sarfraz Hashim, Xie Yuebo, Fiaz Ahmad, Chaudhry Arslan, Muhammad Saifullah

**Affiliations:** ^1^Department of Hydrology and Water Resources, Hohai University, Nanjing, Jiangsu 210098, China; ^2^College of Engineering, Nanjing Agricultural University, Nanjing, Jiangsu 210031, China; ^3^Department of Agricultural Engineering, Bahauddin Zakariya University, Multan 60800, Pakistan; ^4^Department of Structures and Environmental Engineering, University of Agriculture, Faisalabad 38040, Pakistan

## Abstract

To protect against the environmental pollution, the present research was undertaken to enumerate the Bacterial Technologies (BTs) on the restoration of polluted urban rivers, that is, Fenghu-Song Yang River (FSR) and Xuxi River (XXR). Experimental research accounted for the physiochemical parameters (pH; temperature; dissolved oxygen (DO); chemical oxygen demand (COD); total phosphorus (TP); total nitrogen (TN); and ammonia nitrogen (NH_3_N)) before and after the BT operation. The results declared that the BT is efficient to restore the polluted rivers up to reliable condition. These results were analyzed by using multivariate statistical techniques (principal component analysis (PCA) and cluster analysis (CA)). These techniques interpreted the complex data sets and expressed the point source information about the water quality of these rivers at SA5, SA6, and SB3 under highly polluted regions. For better understanding, water quality index (WQI) was applied to compute the single numeric value. WQI results are evidence of the above results which prove the water quality of both rivers faced under outrageous condition (below 50 WQI scores) before the BT treatment, but, after the treatment, the rivers were restored from fair to good level (above 50 WQI scores) and overall output of these scores was quite similar to detect the point source of pollution. These results described an abrupt recovery of the urban rivers up to reliable condition for aquatic organism and clear effluents from the rivers.

## 1. Introduction

The urban rivers or streams have always been the recipient of sewage water from various sources that have different kinds of the domestic, agricultural, and industrial foreign particles [1]. The odor release from these water bodies stimulates the environmental pollution and the release of odor is often unavoidable due to its natural phenomena [2]. It is commonly known that raw municipal wastewater contains a great number of pathogenic and opportunistic microorganisms, as well as those antibiotic resistants including multidrug resistant, mainly of intestinal origin [3]. The urban river is an elemental source that collects the municipal wastewater with huge amount of sludge [4], the municipal sewage is the mixture of various organic matters, and the decomposition of these matters produces harmful gases that in fact deteriorate the environment and logically created the infection diseases [5]. The deterioration of water quality speedily contributes to water scarcity as a major concern. The Middle Eastern countries are facing the relative asperity of water quality according to various types of factors, including industrialization, nonrenewable water resources, population growth and density, institutional capacity, and economic situation. The constitutional expectation is that villagers have left their primitive residence, due to polluted potable water and food scarcity and skin and waterborne infectious diseases [6, 7]; therefore urban environment is becoming more alarming.

The fresh water resources are depleted because of massive agricultural activities, urbanization, and industrialization [8]. All over the world, the sustainable management of water is a major concern for scientists, politicians, and social workers. In fact, worldwide, the natural processes and anthropogenic activities have been attributed to the surface water quality deterioration, including agricultural land use, hydrological features, sewage discharge, precipitation, and climate change [9–11]. Niemi et al. [12] stated that the human activities, that is, effluent discharge, eroded soil, and agricultural chemicals, are the major factors to deteriorate the surface and ground water. Pathogenic as harmful organisms are concerned in wastewater including bacteria, such as* Shigella* and* Salmonella* viruses and protozoa.

Therefore, to resolve the above issues would be a huge challenge for the planner and policy maker, due to treatment cost and the huge impact of unbridled population acceleration. Here we need a solution that efficiently resolves these critical pollution problems and could be used to rehabilitate the existence systems. Few decades ago, some conventional technologies have been developed and applied to treat the wastewater but these technologies failed due to huge maintenance cost and cannot control the huge effluent impact from various sources [13]. Thus, we need advanced and efficient system that could be helpful for today requirements. BT is an application of bioremediation that uses some beneficial bacterial metabolism to remove nutrients from the water bodies and regenerate the original condition [14]; BT operation cost is low with simple application procedure as compared to other conventional treatment systems [15]. Temperature is a major concern and is directly effective in the process of the degradation of the substances. In addition, beneficial bacteria are usually hired to vitiate pollutants or nutrients into simple, nontoxic substances and produce suitable effluents. BT has been successfully implemented to recover the lakes pollutant [13], restore polluted rivers, and assimilate effluent of wastewater treatment plant. It extends for the purification of polluted rivers and streams and also meeting the requirement on the standards of wastewater effluents without building massive structures as compared to the other conventional methods as needed in wetland construction [16].

The largest microbial community component based on bacteria in all biological wastewater treatment operations and quantity of these species is frequently encountered in the range of 106 bacteria/mL of wastewater [17]. These results are composed of complex and large data matrix of physicochemical parameters, which are usually interpreted with some meaningful techniques. The applications of multivariate statistical techniques, such as PCA, CA, factor analysis, and discriminant analysis, are helpful for interpretation of complex data frame into understandable form of ecology and water quality status of a county. These robust statistical techniques allow the recognition of the possible ways that can offer valuable tool for the rapid solution for pollution problem and water management as well as an influence on the water systems. Therefore, this paper briefly discusses practical implementation of BT on urban polluted rivers and argues with multivariate techniques and water quality index that BT is simple, affordable, and efficient for restoring polluted water bodies that directly or indirectly are a cause of environmental pollution.

## 2. Materials and Methods

### 2.1. Study Area and Sample Analysis

#### 2.1.1. Site A

**Figure 1:**
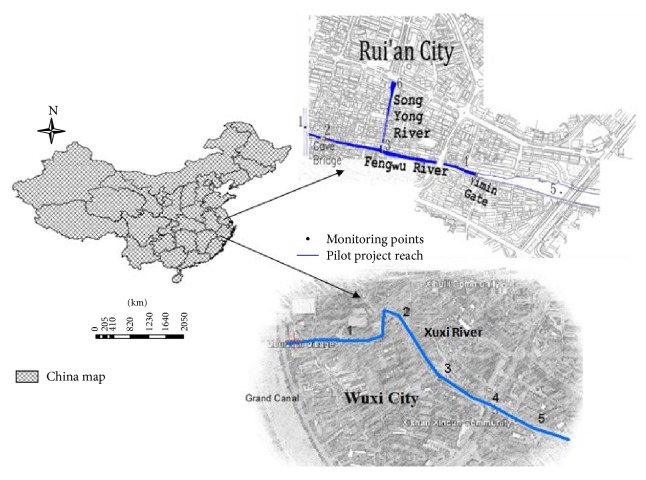
Study area and experiment site.

Fenghu (FH) and Song Yang (SY) Rivers are selected, FH River is placed on the tail of the SY River, and both of them are situated in Wenzhou (Rui'an) city (120°39.13′E and 27°46.49′N), Zhejiang Province, China. Most of the Wenzhou area is placed under the typhoon zone, and the FH River is taking water from Wenruitang and Liangmian River. The SY River is starting from cave bridge and falls directly into the FH River. The SY river length is about 280 m with the average breadth being 5–18 m and 1–3 m water depth, and FH river length is about 740 m with the average breadth being 6–15 m and 1 m water depth. For experiment monitoring and sample collection, the selected length from both rivers was divided into six points as 1 to 6 (Figure 1). The appearance of the river water color was blackish or greenish, and bubbles were blowing on the surface of water. These rivers are situated under the commercial and highly polluted area, and almost 2000 m^3^ sewage water enters into them. Therefore, the average depth of sediments is 0.1 m and the rivers water quality was unsuitable for any purpose.

#### 2.1.2. Site B

Earlier the name of Xuxi River (XXR) was Shaoxiangbanghe and it is situated in Wuxi city, Chang Nan District of China, as geographically (31°56.29′N and 120°28.14′E). Its upper stream starts from the Jing-Hang main canal and travels towards the ancient small canal. The selected river length is 1360 m with 4.5 m of upstream surface average width and about the average depth of 1.4 m. River is under north subtropical humid zone and is marked by muddy sediments. This zone is facing four distinct seasons with the phenomenon of climatic influence circulation.

In both cities of the selected sites, the cause of awful environment is the nonexistence of sanitation facilities for the community. This information was collected from the previous data that most of the quality parameters were retrogressed as compared to Class III of the Chinese National Standard (CNS) for Surface Water Quality (GB2828-2002) [18]. On the basis of current surface water quality standard, the rank of water quality in the source section was determined to be graded V (Table 1). Class V shows the worst (poor) and least water quality standard enlisted by the Chinese National Standard board. For any purposes, the river water is extremely unsuitable. Hence, this site selected for small urban rivers is in the worst category of Class V in China.

### 2.2. Bacterial Implementation

Bacterial Technology (BT) applies in a simple way; its procedure is held under three kinds of material as Bacterial Clusterization (BC), Nature Liquid, and biological filter media. BC is an important material that has a mixture of three types of ingredients as beneficial bacteria (Bacilli,* Bacteroides*, brown-rot spindle, and Lactobacillales, denitrifying with 6 : 4 : 3 : 4 : 3), mixing medium (catalyst process as glucose, sucrose, cellulose liquid, yeast cream, liquorice root, magnesium sulfate, dipotassium hydride, mannitol, tartaric acid (Na; K), folic acid, and ammonium nitrate), and water as in the ratio 4 : 3 : 3, respectively [19]. The mixing ratio represents that it is harmless and has no adverse effects. Nature Liquid (NL) is the mixture of trace element, multiple enzymes, humic acid, amino acid, vitamins, and composition of each adequate substance on judgment. Biological filter media are used on a domestic level as the gap string filter media.

By implementation of BT on FSR, the 100 kg plastic drum was trained, mixed, and injected on the banks of the river as a proportion of the bacterial agent base (3 : 5 : 17) of aerobic and anaerobic cultivated bacteria for testing the suitable condition of the river water quality. Expanding culture of the diluted bacterial agent injected directly into the sediment at point “1” adopts a quincunx location method for monitoring positions. For successful results, a total of 12.9 tons of bacterial amount were employed on the FSR. 0.05% (bacterial agent) of bacteria were injected first time on November 18 to 20, 2011, and afterward 25, 27, and 30 as 0.025%, 0.02%, and 0.04%, respectively. Three times of the bacterial proportion were added on upstream station “1” as the specific bacterial quantity of 780 kg, 360 kg, and 560 kg, respectively. This site was monitored with the specific interval as 1, 2, 3, 6, 7, 8, 9, 10, 13, 14, 16, and 17 days during experiment and the random data collection at least 4 to 7 times in each month up to July 2012. The data was collected in almost 9 months.

**Figure 2:**
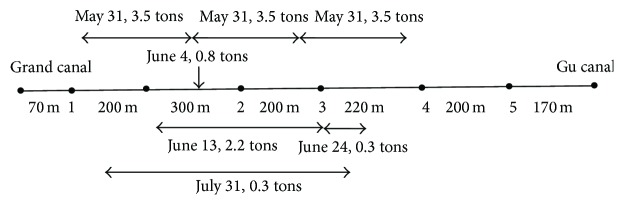
Schematic diagram of XXR and sampling points during BT operation.

By implementation of BT on XXR, a total of 11.1 tons of bacterial amount were employed on the selected points of the river (Figure 2). As the bacterial agent used effectively works under relatively constant and slow flow of velocity, an artificial weir was installed at the end of the river reach which is the small wood bridge to stop the sludge. It was technically built at about 50 cm height above the water surface level in order to extend the hydraulic retention time. This experiment was conducted at the end of May 2009. The XXR site data were collected with specific interval as shown in Figure 2 during the experiment and the data was randomly collected 4 to 7 times in the months after the operation of the BT.

### 2.3. Samples Collection and Pretreatment

The sampling network was arranged to cover the complete range along the inlet and outlet points of the rivers and determined the dominant point sources that have an impact on the water quality. Both of the sites are located under the area of population and industrialization, so the samples were collected from various depths (0.5 ft and >1 ft), at each monitoring station. The samples were collected at 8:30 AM to 4:30 PM during the period of the experiment and physiochemical parameters of temperature, color, pH, DO, COD, TN, TP, and NH_3_-N were collected on the specific monitoring points of both rivers (Figure 1).

To determine the water quality, the samples were preserved in polyethylene bottles and stored in insulated ice cooler delivered to the laboratory on the same day. All the samples were saved at 4°C until the analysis and processing (ISO 5667-6, 1990; ISO 5667-2, 1991; ISO 5667-3, 1994). Then each parameter was tested in laboratory. The test methods were applied to determine the temperature (*T*), pH; and potassium dichromate oxidation for COD. Total phosphorus (TP), ammonia nitrogen (NH_3_-N), and total nitrogen (TN) are commonly associated with wastewater. The growth of algae in large quantities can result from these nutrients. Also, depletion of oxygen concentration is caused by the growth of the algae matter. Therefore, the removal of these nutrients in wastewater becomes a critical part in effective water quality management for rivers.

### 2.4. Numerical Calculations for Data Treatment

All numerical calculation was analyzed by using Excel-2007 and xlstat-2014 software. Multivariate analysis of data set from the urban rivers' water quality was performed through PCA and agglomerative hierarchical clustering (AHC) technique [20]. The WQI approach was used for the better understanding of the water quality, before and after the bacterial restoration of the urban rivers.

#### 2.4.1. Principal Component Analysis (PCA)

PCA has the function of converting the original variables into new uncorrelated variables (axes), which are linear combinations of the original variables and lie along the directions of the maximum variance. PCA provides an objective approach to find this type of indices. So the variation in the data can be accounted for as concisely as possible [20]. PCA provides the information of the meaningful parameters which represents the whole aid data reduction and data set interpretation and summarize the correlation among constituents in the water with minimum loss of the original information. The first principal component loading displays most variance in the observed data, while each of the following components represents progressively less variance. PCA has been used in many regions for the understanding of the water quality [21, 22] and it provides the important information for the data interpretation.

Since the enormous variables of the data have been measured before and after the experiment related to the restoration of the urban rivers. Most of the data is raw and it is very hard to understand the environment conditions and historical changes to the data. Therefore, these techniques are useful, when large frame of data needs to be analyzed for the target area. In this research work, PCA was used to calculate the Pearson (*n*) correlation among components in the water samples of both urban rivers taken before and after the treatment.

#### 2.4.2. Agglomerative Hierarchical Clustering Analysis (CA)

The CA is an unsupervised technique which involves measuring the similarity or dissimilarity of the distance between the concerned objectives to be clustered. The resulting clusters of the objects should then exhibit high internal homogeneity and high external heterogeneity. Hierarchical clustering is the most common approach that is used for the instinctive similar relationship within the entire data set of the samples and illustrated by the dendrogram [20]. For the visual summary of the clustering process, dendrogram represents the agglomerative group drawing and its approximity, with a dramatic reduction in the dimensionality of the original data. The Euclidean method represents the analytical values usually used for the dissimilarity between two samples and distance [23]. In this research, the agglomerative hierarchical clustering technique was applied to the normalized data frame of both sites by means of Ward's method and spatial similarity detected by using squared Euclidean distance method, for the stations grouping under the monitoring of the experiment.

#### 2.4.3. Water Quality Index

The surface water quality could be an intricate process undertaken to determine the different concerned parameters accomplished of large stresses capable of overall water quality of the river [24]. For pollution abatement, environmental management, and decision making, the water quality assessment plays a fundamental role. Therefore, to determine the water quality assessment, a water quality classification model should be developed firstly, and then we should apply the model to calculate the class of the water quality based on the index values. Multi-index assessment model was established to obtain an appropriate value of the water quality. Once the data are collected from the experiment, they further need to translate into simple and effective interpreted form. Water quality indices are the best tool to represent the data in a simple and understandable format [25].

Many sanitation and environmental foundations have formulated numerous water quality indices which are used all over the world to easily judge and better understand the overall water quality of the water body within a particular area promptly and efficiently [26]. Numerous indices are based on various techniques that summarized the result in single number: for example, the Canadian Council of Ministers of the Environment Water Quality Index (CCME-WQI), US National Sanitation Foundation Water Quality Index (NSF-WQI), Oregon Water Quality Index (OWQI), and British Columbia Water Quality Index (BCWQI) [25–28]. These indices are formulated with the specific weighting ratio with the comparison of all water quality parameters with the standard values and provide a single numeric value to the source water quality. In this research, the index was used to evaluate water quality of both urban rivers during the BT operation that was formatted by the US National Institute of Health for water quality ranking in the early 1970s. Using the following equation, the US National Institute of Health formed water quality grading:

(1) $$\begin{array}{c} \text{WQI}=\overset{n}{\underset{i=1}{\sum }}w_{i}q_{i}, \end{array}$$

where WQI stands for the water quality index, “*w*_*i*_” is weighting ratio, “*q*_*i*_” is the value acquired according to the relevant parameters, and “*n*” is the number of parameters. For calculating the values of the index, Table 2 demonstrates the ranking of the water quality. For the present research, the variable weighting ratio was calculated from Table 3 and classified each station based on specific rank as shown in Table 2.

## 3. Results

The selected urban rivers were situated under the appalling environment and the rivers' conditions were awful before the operation of BT and they were located in commercial area; also industries were located along the side of the rivers. The huge amount of sewage loading was directly entered into these rivers. There was no sanitation facility to control or dump the municipal sewage to fall into rivers, so that sewerage made a part of urban rivers without any preliminary treatment. Therefore, there is an urgent need to treat these urban rivers for pollution abatement and to rehabilitate the aquatic environment of the rivers.

**Figure 3:**
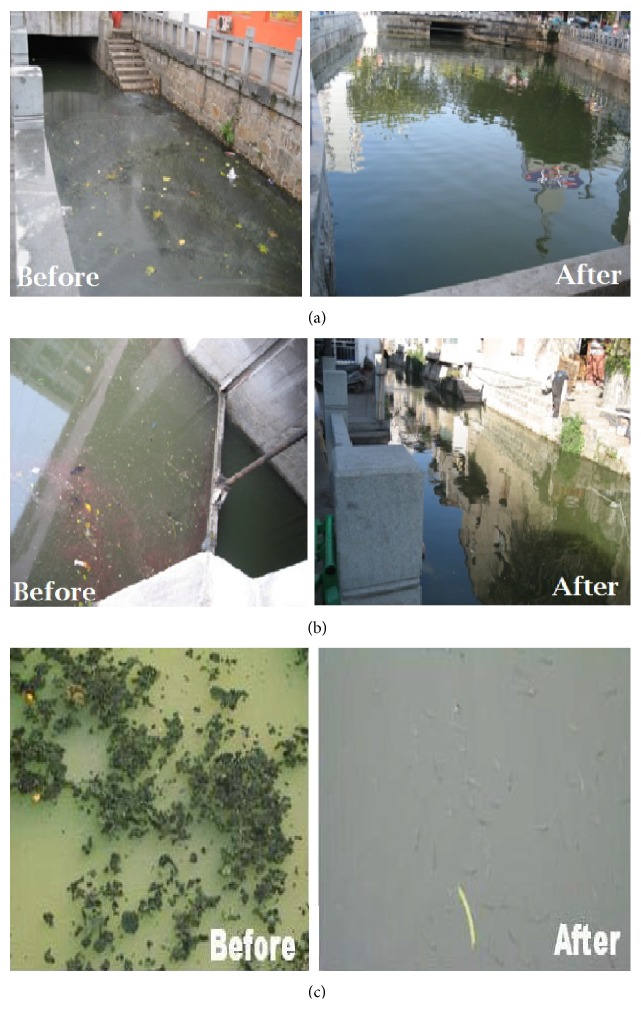
(a) Contrast diagram of Fenghu River. (b) Contrast diagram of Song Yong River. (c) Contrast diagram of Xuxi River.

BT has applied in three sessions (long summer to short winter) of the year on the selected rivers and water samples were collected during the treatment of the rivers. The range, mean, and standard values of each parameter are in Table 4, after determining the values, compared with the improved efficiency of nutrients before and after bacterial action which was 79%, 74%, 68%, 70%, and 65% of DO, COD, TP, TN, and NH_3_-N, respectively. From the results, the DO was the most critical parameter for aquatic life of rivers that have maximum efficiency. To protect from the environmental pollution, DO also plays an important role in respiration of aquatic animals. Improved efficiency of TP, TN, and ammonia nitrogen also has favorable results. For more consideration, the color and algae also recovered as shown in Figures 3(a), 3(b), and 3(c).

### 3.1. Numerical Evaluation of the Experiment

**Figure 4:**
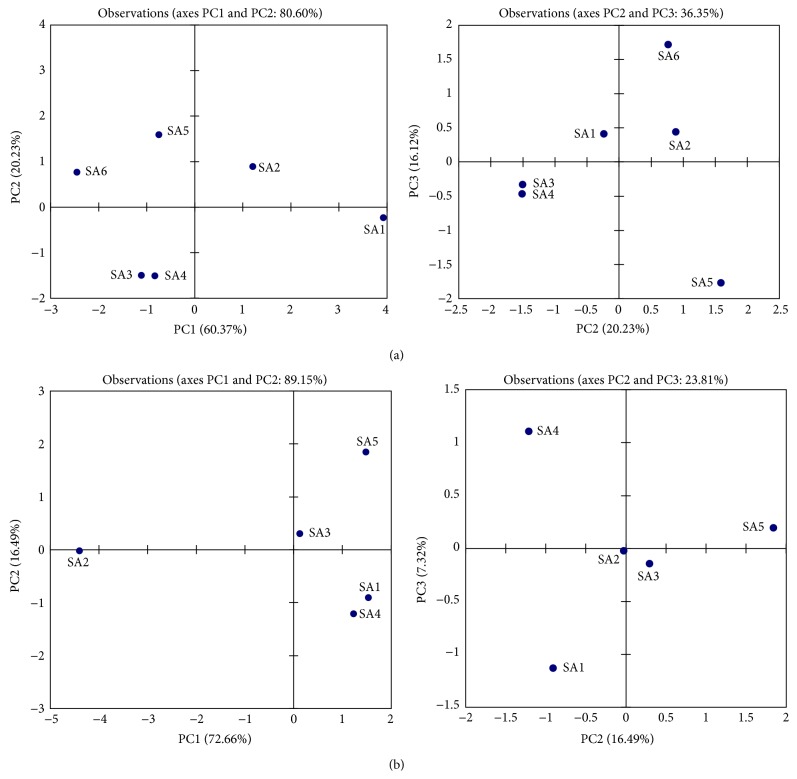
Scores of principal components: (a) FSR, (b) XXR.

The fundamental statistics of these restoration experiments contained 1485 total water samples (11 monitoring points × 4 data frequencies × 5 replications × 9 months) as before and after the BT operation and all data is summarized in Table 4. The data were collected during a period of 9 months and each station of the site was monitored with spatial as well as temporal variation. The PCA results are formulated chemical components based on Pearson (*n*) correlation matrix (Table 5). Three components of PCA analysis represent 96.72% and 96.47% of the variance in the sampling data of FSR and XXR, respectively (Figure 4). The eigenvectors classified the physiochemical parameters into three groups based on the PCA values: the first group contained temperature and pH, the second has DO and COD parameters, and the remaining TP, TN, and NH_3_N are in the third group.

**Figure 5:**
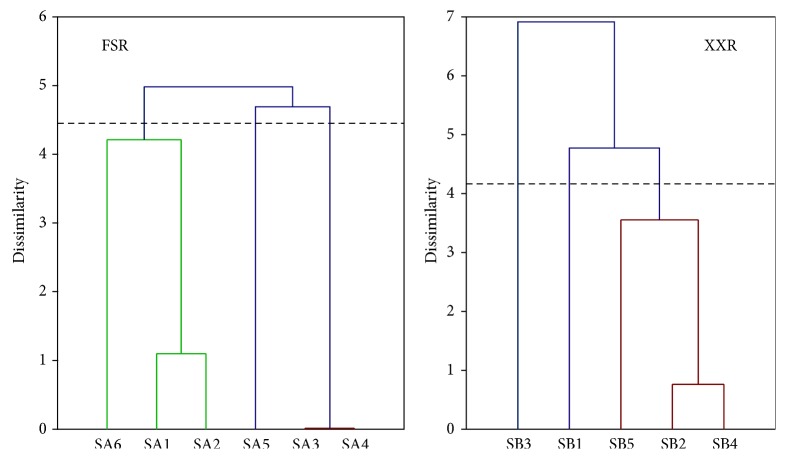
Dendrogram for agglomerative hierarchical clustering analysis.

Agglomerative hierarchical clustering analysis was applied on both restoration sites of the water quality, to determine the spatial dissimilarity grouping for the monitoring stations along the rivers. The spatial variation results indicated three significant clusters for six sampling stations on FSR site, as stations 1–4 have two clusters such as (1-2) and (3-4) which shows low mutual dissimilarities as compared to the third cluster or (5-6) stations and on XXR, the five sampling stations into three statistical clusters as (2, 4, 5) have low mutual dissimilarity as compared to (1, 3) station (Figure 5). From these results, two main objectives solved for the assessment of the water quality. On XXR, the 3rd station was placed under the area of industrialization and there is a main horrific point of pollution along the river and the FSR; downstream stations were located under polluted and worse condition and all the domestic and industrial wastes were received there.

**Figure 6:**
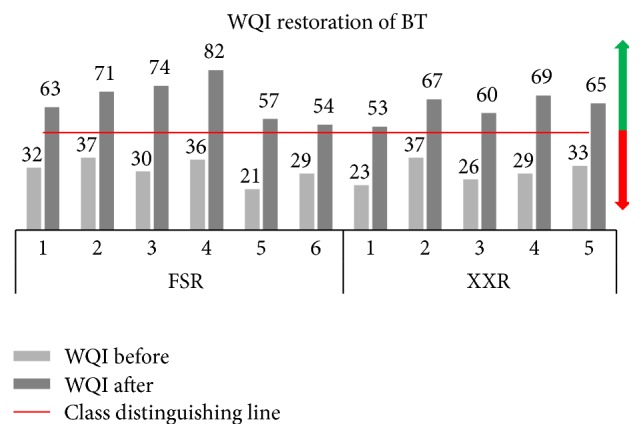
Water quality index on restoration of FSR and XXR.

For a comparative analysis of before and after the restoration of the rivers, WQI was applied and evaluates the water quality assessment of the rivers. The results declared that the selected rivers' water quality was poor (Class V) in both sites, before the treatment process as explained in Table 2; their environment was polluted and may cause deterioration of the environment from these urban rivers. After the BT operation, the water quality was restored up to reliable condition (Figure 6). To distinguish the classes of WQI scores, a red line was drawn for the better clarification and the scores below the line represent the poor class as red arrow shows the worse indication, but as to grow up the WQI scores, it declares the “fair to good” water quality as green arrow indicates that the BT operation is helpful to mitigate the pollution up to reliable condition.

## 4. Discussion

### 4.1. Urban Rivers Chemistry

All the physiochemical parameters were collected during the BT experiment. The minimum and maximum values of water samples are presented in Table 4, and the results from both sites are compared to the values of Chinese standards of surface water as recommended for the permissible limits of Classes I–V. Temperature is the main parameter to employ BT at any location; because the temperature increases, molecules move faster, enzymes speed up metabolism, and cells rapidly increase in size. But, above a certain value all of these activities are proceeding at such high rates, enzymes start to denature, and the total effect is detrimental. Cellular growth ceases. Therefore, in these concerned sites of urban rivers, the experiment was performed under the temperature range from 15°C to 30°C.

### 4.2. Site Station Grouping and Spatial Dissimilarity

To normalize the complex data frame of both sites, PCA was applied and differentiated by hierarchical cluster technique to evaluate the comparative composition pattern among the samples taken from the monitoring stations. In view of macroscopic consideration, all the physiochemical parameters have the same behavior as the high concentration of toxic nutrients in whole urban rivers, but the light variation could exist on the loading pollution with the temporal effects. For rapid assessment, CA technique is useful for each cluster of the whole network to evaluate the water quality. It is the evidence that both restoration sites are classified based on sampling station along the whole river and adequately divide these stations into specific optimal manner for BT evaluation.

The DO is the major concern under these restoration sites; the key parameters rehabilitate the aquatic environment and are also helpful to degrade the toxic metals. The negative loading of ammonium nitrogen was observed, whereas the strong positive loading on DO has up to 79% efficiency. Thus, the main advantage of the BT is that the bacteria have self-reproduction property that addresses the polluted and harmful particles from the urban bodies. The PCA trend obtained was also helpful for the analysis of the raw data set. The second component shows the 20.23% in FSR and 16.49% in XXR of the strong loading of DO and COD in the total variation. The third component shows the 16.12% and 7.32% of the total variance in FSR and XXR, respectively, for the higher values of TP and TN are above the allowable limits of the Chinese standards values for the surface water quality.

The AHC results based on PCA scores clarify the abnormality in the dendrogram of both sampling sites with each monitoring station, which make the cluster groups with identical effluents variance from the various aspects of parameters. In FSR, the cluster group (5-6) was placed downstream under the number of nonpoint sources as agricultural, industrial, and domestic sewage. Besides the mutual dissimilarity among other clusters (1-2) and (3-4) have relatively low pollution status as compared to correspondence. Similarly, cluster group is verifying the pollution status in XXR upstream at SB1 and at the monitoring station SB3, the nonpoint sources take part in increasing the pollution due to anthropogenic activities and commercial area. The comparative results from both sites declared that monitoring stations (SA3, SA4), (SA1, SA2), (SA5, SA6) for FSR and (SB2, SB4), (SB5), and (SB1, SB3) for XXR have relatively low, medium, and high pollution regions, respectively. It implies that for rapid evaluation of the water quality only one station from each cluster may be used to describe the spatial evaluation of the overall network. For example, Tabata et al. research investigated the Ariake Sea, Japan [29]. They have interpreted 11 water quality parameters by using PCA, to determine the organic pollution level and seasonal changes. Kazi et al. used multivariate techniques on Manchar Lake, Pakistan [20]. He interpreted PCA and CA with 36 water quality parameters to determine the deterioration cause of the lake water quality and anthropogenic activities impact on the Manchar Lake. Tabata et al. also evaluate the organic pollution and seasonal variation of the Ariake Sea through PCA and applied AHC to clarify the PCA scores. Therefore, it is evident that the AHC is a useful technique to provide helpful classification for the assessment of the whole region's water quality in an optimal pattern. Thus, the total number of monitoring stations and cost of the network could reduce without fixing of any significant outcome.

For better understanding and single numeric value, WQI was applied on both experimental sites which aim to reduce the complex data frame into optimal manner and enable interpretation of the monitoring data into a single numeric format. WQI results are revealed in Figure 6, which describes the pollution status of each monitoring station as before and after BT implementation. The WQI scores of FSR at 5 and 6 stations show that the values vary from 21 and 29 to 57 and 54 before and after the BT operation, respectively. Similarly, the WQI results of XXR site are expressed in Figure 6. It shows that the upper stream and middle of the river are under worse conditions and the WQI scores are varying from 23 and 26 to 53 and 60, respectively. It is evident from the above results that the river region is under an appalling environment with outrageous condition before the BT operation. The comparative results, before and after the BT operation, are quite similar to the above results as generated from AHC. Thus, the efficiency to restore the pollution from before to after BT could help to employ the amount of bacterial agent at any instant. Therefore, WQI was applied to evaluate the whole network of both urban rivers.

### 4.3. Cost Benefit Ratio Based on Conventional Technologies

Temperature is the major concern under the metabolism process of the bacteria, with higher temperature (20–35°C) during the summer from May to September and lower temperature (5–18°C) in the winter season from December to February. From the results, it is observed that the BT can perform the best outcome under 15–30°C temperature. The plus advantage of BT is that it does not need to demolish existing system. Therefore, new or existing systems could be easily integrated with the BT for continuous enhancement in the operation. BT has no effect on the natural environment because it does not involve the use of chemicals. Therefore, it is helpful for friendly ecology. All other issues, such as construction and high maintenance costs as other conventional systems have been operated that made a huge burden to sanitation foundation, government, and policy makers. Recent discussions on the urge for green technologies and climate change compel all the countries to use reliable technologies that are reasonably sustainable. Sustainability stands to mean activities or development that meets the needs of the present without distrusting the needs of the future generation.

In view of these revolutionary calculations, the adoption of BT becomes the most convenient approach for developing countries as has been concluded [19]. The cost of treating the tons of wastewater is about 241≈321$ which WWTPs is being built, under construction and already built, the operation cost is between 0.12 and 0.22$ per ton [15]. According to this statement, the municipal sewerage operation system needs to spend above 16 × 10^7^$ on a single attempt. If the pipe network of municipal administration is built, the amount of total cost will exceed 32 × 10^7^$, and the operational cost is raised up to 4 × 10^7^$ annually. Therefore, the addition of bacteria to treat the sewerage wastewater, the one off investment cost is only 65$ and this method is simple, easy to operate, and affordable. For the long term, there is no need of maintenance and artificial dregs up to 10 years [15]. In addition, the existing sanitation systems are deteriorating due to many-imperfection care. So BT has the ability to restore these systems due to its self-purification property. Similarly the maintenance cost of the sewerage system is unfavorable due to economic collapse. So we can prefer BT due to its simplicity, affordability, ecofriendliness, and adoptability for any scale of system and existing program.

## 5. Conclusions

This research emphasized that the BT offers an innovative technique which provides an ingenious solution for the rehabilitation of the polluted urban streams. The results declared that both rivers were under outrageous condition of water quality that directly affects environment ecology, while after BT operation the worse condition of the urban rivers was rehabilitated. In this research, various multivariate techniques were applied to determine the spatial and temporal variations along the rivers. This study demonstrates interpretation of the problems of the complex data set through multivariate techniques because chemometric research enables us to discuss the similarities and dissimilarities along the observing stations among the variables that could not be clearly visible for assessment of the analytical data in a table. From overall results it is concluded that BT is efficient due to its simplicity and being economical, affordable, and reproducible on any scale of the operation. Hence, it is helpful to find reasonable, reliable, and efficient solution to the various future water pollution problems all over the world.

## Figures and Tables

**Table 1:** Water quality parameters of both scenarios and Chinese National Standard.

		Monitoring project						
Sampling time		Water temperature °C	pH	DO	COD	TP	TN	NH_3_N
				mg/L	mg/L	mg/L	mg/L	mg/L
National Standard GB3838-2002		Class V index	6–9	2.00	15.00	0.0	2.00	2.00

FSR	14:40	16.1	7.5	2.5	10.90	0.96	14.80	10.60
	River water quality class		—	V	V	Inferior V	Inferior V	Inferior V

XXR	16:00	27.2	8.77	2.81	12.10	0.82	14.90	11.20
	River water quality class		—	V	V	Inferior V	Inferior V	Inferior V

**Table 2:** WQI criteria of ranking.

Rank	Excellent	Good	Fair	Marginal	Poor
WQI value	91–100	71–90	51–70	26–50	0–25

**Table 3:** Weighting ratio of water quality parameters for WQI.

Parameter	Temperature	pH	COD	TP	TN	NH_3_N	DO
Wi	0.19	0.11	0.15	0.12	0.13	0.13	0.17

**Table 4:** Water quality data of FSR and XXR experiment data.

Parameters	GB2828-2002 (CNS)		Fenghu-Song Yang River data						Xuxi River data				
			SA1	SA2	SA3	SA4	SA5	SA6	SB1	SB2	SB3	SB4	SB5
pH	6.5–8.5	Mean	7.3	7.4	7.2	7.3	7.4	7.6	7.3	7.7	7.5	7.6	7.5
		Standard deviation	0.27	0.27	0.27	0.27	0.27	0.24	0.28	0.23	0.26	0.23	0.26
		Minimum	7	7	7	7	7	7	7	7	7	7	7
		Maximum	7.5	7.5	7.5	7.5	7.5	7.5	7.5	7.9	7.8	7.9	7.8

DO (mg/L)	2	Mean	1.69	1.69	1.83	2.14	2.46	2.39	0.65	0.77	0.76	0.63	1.06
		Standard deviation	1.08	0.9	0.9	1.16	1.38	1.39	0.21	0.38	0.58	0.36	0.57
		Minimum	0.4	0.4	0.5	0.5	0.5	0.5	0.4	0.2	0.1	0.2	0.1
		Maximum	3.3	2.7	2.8	3.3	4.1	4	1.2	1.6	1.7	1.6	1.8

COD (mg/L)	15	Mean	70.37	72.24	59.7	59.01	59.01	58.53	13.63	10.73	13.3	12.9	13.73
		Standard deviation	49.29	52.45	35.22	40.32	35.2	30.87	1.44	2.34	1.54	1.41	1.27
		Minimum	28.4	29.1	29	24.7	24	24	11	5.6	11.2	9.7	11.3
		Maximum	147.7	150.1	117.2	122	123	109	16.7	15.6	16.8	15.4	15.6

TP (mg/L)	0	Mean	0.91	0.84	0.83	0.76	0.84	0.78	1.23	0.72	1.14	1.13	1.27
		Standard deviation	0.57	0.5	0.47	0.47	0.42	0.37	0.26	0.13	0.16	0.17	0.15
		Minimum	0.14	0.14	0.13	0.15	0.4	0.4	0.7	0.5	0.87	0.83	1
		Maximum	1.86	1.57	1.44	1.47	1.45	1.3	1.66	1.02	1.44	1.46	1.49

TN (mg/L)	2	Mean	29.31	23.17	24.44	23.01	25.77	27.31	25.31	21.17	22.44	21.01	21.77
		Standard deviation	3.57	4.45	2.18	2.27	1.36	3.58	3.47	4.28	2.16	2.25	1.36
		Minimum	19.2	17.5	17.5	17.4	20.3	19.2	19.2	13.5	18.5	16.4	19.3
		Maximum	37.1	36.2	29.6	26.8	31.6	34.1	32.1	28.2	26.6	24.8	24

NH_3_N (mg/L)	2	Mean	14.21	13.87	11.49	10.59	11.44	12.02	17.8	10.65	15.59	15.62	17.83
		Standard deviation	9.77	10.23	9.99	11.5	10.44	7.9	3.08	1.49	1.82	1.17	1.31
		Minimum	2.42	2.01	2.04	0.87	1.76	1.43	11.5	8.35	12	13.3	15.9
		Maximum	27.3	26.9	25	26.7	26.7	24	23.6	14.4	20.1	18.1	20.1

Temperature (°C)	15–20	Mean	16.5	16.43	16.43	16.46	16.7	15.5	25.9	27.2	26.4	26.2	26.2
		Standard deviation	0.32	0.36	0.29	0.34	0.35	6.23	3.33	3.49	3.38	3.39	3.45
		Minimum	16.2	16	15.9	16	16.4	15.2	25.8	26.6	25.8	25.7	25.8
		Maximum	17.1	16.9	16.8	16.8	17.4	16.7	26.5	28	27	27.1	27.5

**Table 5:** Eigenvalues and vector on correlation matrix of water quality variables in FSR and XXR.

Parameters	FSR components			XXR components		
	1	2	3	1	2	3
pH	**0.727**	−0.089	−0.076	**0.628**	−0.086	−0.186
Temperature	**0.463**	−0.345	0.035	**0.842**	−0.324	0.112
DO	0.143	**0.517**	−0.062	0.317	**0.928**	−0.023
COD	0.144	**0.837**	0.162	0.213	**0.532**	−0.864
TN	0.063	−0.093	**0.745**	0.171	−0.271	**0.868**
TP	0.157	−0.053	**0.827**	0.147	−0.138	**0.914**
NH_3_N	0.241	−0.104	**0.771**	−0.139	0.034	**0.875**
Eigenvalue	4.23	2.42	1.13	5.09	1.15	0.51
Variability %	60.37	20.23	16.12	72.66	16.49	7.32
Cumulative	60.37	80.60	96.72	72.66	89.15	96.47
